# Neurologic Complications of Cancer Immunotherapy

**DOI:** 10.3390/curroncol30060440

**Published:** 2023-06-19

**Authors:** Aseel N. Alsalem, Leslie A. Scarffe, Hannah R. Briemberg, Ashley E. Aaroe, Rebecca A. Harrison

**Affiliations:** 1 Division of Neurology, University of British Columbia, Vancouver, BC V6T 2B5, Canada; aseel.alsalem@alumni.ubc.ca (A.N.A.); leslie.scarffe@mail.mcgill.ca (L.A.S.);; 2 Department of Neuro-Oncology, University of Texas MD Anderson Cancer Center, Houston, TX 77030, USA; 3 Division of Medical Oncology, BC Cancer, University of British Columbia, Vancouver, BC V5Z 4E6, Canada

**Keywords:** immunotherapy, neurologic complications, checkpoint inhibition, CAR-T cells

## Abstract

Immunotherapy has revolutionized cancer treatment over the past decade. As it is increasingly introduced into routine clinical practice, immune-related complications have become more frequent. Accurate diagnosis and treatment are essential, with the goal of reduced patient morbidity. This review aims to discuss the various clinical manifestations, diagnosis, treatments, and prognosis of neurologic complications associated with the use of immune checkpoint inhibitors, adoptive T-cell therapies, and T-cell redirecting therapies. We also outline a suggested clinical approach related to the clinical use of these agents.

## 1. Introduction

The introduction of cancer immunotherapy has led to a paradigm shift in cancer treatment due to improved survival and more favorable safety profiles compared to traditional chemotherapy [1,2]. Since the first immune checkpoint inhibitor (ICI), ipilimumab, was approved in 2010, additional agents have been introduced into standard oncologic care. In recent years, other forms of immunotherapy, including adoptive cell therapies, have also entered the clinical arena.

Under physiological circumstances, immune checkpoints play a role in the maintenance of self-tolerance [3,4]; the dysregulation of these pathways by cancers is thought to be an important mechanism of immune evasion [5]. The most widely studied immune checkpoint inhibitors are cytotoxic T-lymphocyte-associated antigen 4 (CTLA4) inhibitors and programmed cell death protein 1 (PD-1) and ligand 1 (PD-L1) inhibitors. Both of the former function to disinhibit T-cell activity at various stages, leading to enhanced T-cell activity in tissues and the tumor microenvironment at different stages [5]. CTLA-4 inhibitors are thought to exert their effect in the T-cell priming phase within lymphoid tissue, and PD-1/PD-L1 inhibitors act on T-cells in the tissues peripherally [6]. The disinhibition of the immune system leads to various adverse effects deemed immune-related adverse events (irAEs), but the exact mechanism of toxicity is not fully known. The biologic underpinnings of these adverse immune responses are likely complex—there is evidence for T-cell infiltration into target tissues, the generation of autoantibodies suggesting an additional B-cell mediated immune response, as well as increased levels of circulating inflammatory cytokines [6]. 

Adoptive T-cell therapies involve ex-vivo purification, modification, and expansion of autologous T-lymphocytes that are then transfused into the patient. Current approaches include T-cell receptor (TCR) therapy and chimeric antigen receptor (CAR) T-cell therapy. Of these, only CAR T-cell therapies have Food and Drug Administration (FDA)-approved indications to date, though other mechanisms of cell therapy are being explored in the clinical trial setting. CAR-T cells are autologous T-lymphocytes genetically manipulated to introduce an artificial receptor into the immune effector cell. These involve a modified T-cell receptor comprised of an antigen-binding fragment that recognizes a cell surface protein on the tumor cell. This fragment is conjugated to the T-cell receptor CD3 zeta intracellular signaling domain with the addition of a co-stimulatory domain, allowing these T cells to become activated and take upon a cytotoxic phenotype upon encountering their antigen [7]. 

Bispecific T cell Engager antibodies (BITEs) have overlapping toxicities with CAR T cells. They are comprised of fragments of two different antibodies, typically one that binds CD3 co-receptors on T cells and another that binds tumor cell antigen [8]. The best characterized BITE is blinatumomab, which targets CD19-positive B cells in B-cell lymphoma /leukemias and also binds CD3 on T cells. Thereby, CD3-positive T cells are redirected to CD19-positive tumor cells and engaged to lyse the latter [9,10]. Both CAR T cells and bispecific antibodies result in patient T cells directing a cytotoxic response to cells expressing the chosen antigen.

These therapeutic advances have been accompanied by novel treatment-related toxicities. Neurologic toxicities, in particular, have received significant attention due to their potential for significant morbidity. With the growing number of immunotherapies in clinical trials and standard oncologic care, a clinical fluency in these conditions amongst oncologists, neurologists, and those involved in the care of these patients is increasingly important. This paper aims to review the clinical features, diagnosis, and management of neurologic irAEs associated with the use of immune checkpoint inhibitors, CAR T-cell therapies, and BITEs.

## 2. Neurotoxicities Associated with Immune Checkpoint Inhibitors

### 2.1. Central Nervous System Complications

#### 2.1.1. Encephalitis

The estimated proportion of encephalitis among patients treated with an ICI was 0.86% in one pharmacovigilance study [11]. The clinical phenotype of ICI-related encephalitis is often non-specific. Clinically, the majority of patients present with altered mental status, and around 30% have seizures [12,13,14,15]. Two distinct clinical phenotypes can occur: a diffuse meningoencephalitic picture characterized by fever, headache, and altered level of conscious, or a focal encephalitic picture presenting with neuropsychiatric changes, extrapyramidal signs, cranial nerve abnormalities, brainstem, or cerebellum involvement [13,15]. The differential diagnosis includes infectious, inflammatory, autoimmune, or paraneoplastic encephalitides.

Neuroimaging findings are variable, with 51% having a normal MRI [12]. Compared to patients with HSV-encephalitis, patients with ICI-related encephalitis more frequently had a normal MRI [15]. Abnormal MRI findings were more common in patients with focal encephalitis [13] and included T2 hyperintense lesions in the medial temporal lobes, basal ganglia, diencephalon, or subcortical white matter [12,13]. Pachy- or leptomeningeal enhancement has also been reported [14,16]. 

CSF analysis is abnormal in more than 90% of patients [12,13], typically with a lymphocytic pleocytosis and elevated protein. The exclusion of infectious and neoplastic causes is prudent as these CSF findings are nonspecific. Autoantibodies were detected in approximately 50% of cases as reported by Stuby et al. [17] and Marini et al. [12], and 30% of patients in the series by Velasco et al. [13], most commonly intracellular onconeural antibodies such as anti-Hu or anti-Ma. Other autoantibodies reported in association with ICI-related encephalitis include anti-Ri, anti-GAD, anti-NMDAR, anti-CASPR2, anti-CRMP5, and anti-SOX1 [12,13,16,17]. Interestingly, Velasco et al. [13] also found that patients with positive onconeural autoantibodies more frequently had a more aggressive focal encephalitic presentation. This raises the question of whether ICIs play a role in unmasking paraneoplastic syndromes.

Prognosis is generally favorable [12,13], though patient subgroups with ICI-related encephalitis associated with a positive intracellular autoantibody had worse outcomes [13], which may explain the findings of Vogrig et al. [16], who found that 5/8 patients in their case series died from neurologic complications, and 7/8 of their cohort had positive anti-Ma antibodies. Other poor prognostic factors include the presence of a focal syndrome or an abnormal MRI [13]. No data exist on long term cognitive outcomes in this group of patients. 

#### 2.1.2. Meningitis

Meningitis can occur with associated encephalitis, described above, or in isolation. In the latter, patients will often lack the pronounced mental status changes or focal neurologic signs that suggest parenchymal CNS involvement, except if there is associated intracranial hypertension. It is estimated to occur in 0.38% of patients treated with ICIs [11]; however, given the frequency of headache in patients treated with ICIs, the possibility of more frequent low-grade aseptic meningitis should be considered [18]. Clinically, symptoms are similar to those of other causes of infectious and noninfectious meningitis, with fever, headache, neck pain, and nausea, or vomiting dominating the clinical picture [12,19,20]. Symptoms develop after a median of 2 cycles but may occur up to 14 cycles later [20]. Lumbar puncture shows a nonspecific lymphocytic pleocytosis and mild–moderately elevated protein (median 0.87 g/L) [20]. MRI is similarly nonspecific, with about half of cases showing meningeal enhancement [19,20]. Meningitis typically has a more favorable prognosis than encephalitis, with 11 of 13 patients in one systemic review recovering completely [12].

#### 2.1.3. Hypophysitis

The categorization of hypophysitis varies, and hypophysitis is often classified as an endocrine irAE as opposed to a neurologic irAE. Its signs and symptoms are important for neurologists to be aware of however, as its clinical presentation overlaps with that of other neurologic irAEs. Hypophysitis is estimated to occur in 1.79% of patients treated with ICIs, with an almost 300 times higher risk compared to patients not treated with an ICI [11] and occurring a median of 2.3 months after initiation of the drug [21]. This risk is mostly driven by the use of CTLA-4 inhibitors as opposed to PD-1 inhibitors [11,21].

Clinical diagnosis is often challenging, as patients may present with signs of raised intracranial pressure, such as headache, nausea, and vomiting, or visual field deficits, or with symptoms of hormonal deficiency, such as hypothyroidism, diabetes insipidus, or adrenal insufficiency [22,23]. Subtle symptoms such as sinus pressure or fatigue are possible, and hypophysitis may also be identified incidentally on laboratory testing or neuroimaging. Of the endocrinopathies, adrenal insufficiency is by far the most common manifestation of ICI-related hypophysitis [21]. A high index of suspicion should be maintained for patients on an ICI with new onset headache, particularly if the aforementioned symptoms accompany this, and endocrinology consultation is recommended. If hypophysitis is suspected, laboratory testing includes thyroid function tests, adrenocorticotropic-releasing hormone, and follicle-stimulating and -luteinizing hormones. Hypophysitis may also be found in association with other immune-related adverse events [24]. New unexplained hyponatremia, particularly in association with the symptoms noted above or other neurological issues, may also raise suspicion for an immunotherapy-related toxicity. Treatment consists of steroids and the replacement of deficient hormones.

#### 2.1.4. CNS Demyelination

Demyelinating disorders are relatively uncommon complications of ICI therapy, with no significant signal detected in a pharmacovigilance database analysis [11]. This was corroborated in a study by Kelly et al. [25], who found a low prevalence of iatrogenic CNS inflammation, with seven cases of ICI-related demyelinating events among 422 patients, with a prevalence of 0.016%. The majority of the iatrogenic events reported were monophasic. They also found that patients taking an ICI were less likely to present with a relapsing demyelinating disorder compared to patients who experienced immune-related adverse events following TNF-a inhibitor use or vaccination [25]. A systematic review by Oliveira et al. [26] described 23 patients with CNS demyelinating disorders associated with ICI use, including eight cases of myelitis (including one with seropositive NMOSD), four cases of optic neuritis, three with a relapse of known multiple sclerosis (MS), two cases of radiologically isolated syndrome (RIS), and six atypical demyelinating lesions.

ICI therapy has been associated with both new onset demyelinating disease, such as RIS, MS, or acute disseminated encephalomyelitis (ADEM), and relapses of known multiple sclerosis [27,28,29,30]. The clinical presentation is heterogenous, and no neuroimaging finding is specific [11,31]. CSF is typically inflammatory, with oligoclonal banding positivity in approximately 60% of cases [11]. ICI-related demyelinating events are typically monophasic, with the majority of patients achieving partial or complete symptom resolution with therapy [11,26,28]. However, tumefactive lesions with poor response to corticosteroid therapy may occur [29]. Interestingly, patients with a known history of MS may have a more aggressive course, with 4/9 patients experiencing a poor outcome (disability or death) in one analysis [31]. 

ICI-related transverse myelitis may be short-segment [32] or longitudinally extensive [33,34,35,36]. CSF analysis is typically inflammatory, with lymphocytic pleocytosis and elevated protein. Positive oligoclonal banding and an elevated IgG synthesis rate have been reported [11,26]. The majority of cases are seronegative, but cases of aquaporin-4 positive NMOSD [37,38] and paraneoplastic autoantibodies, including CRMP5 [33] and other novel autoantibodies [36,39], have been reported. Approximately 70% patients attain a partial or complete response to variable combinations of steroids, IVIG, or plasma exchange [12,26]. In those who do not, treatments such as rituximab, tacrolimus, and infliximab have been used [40]. These clinical presentations beget the question of whether the ICIs are unmasking a latent predisposition to inflammatory demyelination in some patients. 

Optic neuritis can occur in isolation or in conjunction with other sites of CNS demyelination [26]. It is typically bilateral, painless (as opposed to classical ON in adults), and frequently associated with disc edema. Visual recovery is usually favorable, with partial or complete recovery after the administration of systemic steroids [26,41,42]. MRI may be normal or may show T2 enhancing lesions of the optic nerve. Most cases of optic neuritis are seronegative; however, there is one case of optic neuritis secondary to antibody-positive NMOSD 3 months following ipilimumab and nivolumab adjuvant therapy [43]. 

#### 2.1.5. Vasculitis

In a pharmacovigilance study [11], 100 cases of vasculitis were detected amongst 3619 patients with neurologic adverse effects of ICI, with a crude reporting odd’s ratio of 1.50. They did not specify whether these cases represented central or peripheral nervous system vasculitis. A review of 20 patients with ICI-related vasculitis found that large and medium vessel vasculitis, including giant cell arteritis (GCA), was the most common manifestation followed by central nervous system vasculitis, including four cases of primary angiitis of the CNS (PACNS) [44]. Clinical presentation of patients with ICI-related GCA is similar to idiopathic GCA with transient visual loss, diplopia, headache, scalp tenderness, or jaw claudication [42]. The clinical presentation of PACNS can be nonspecific, with subacute headache, encephalopathy, or progressive focal neurologic deficits [45,46,47]. CSF analysis in PACNS may be normal [45] or show pleocytosis and elevated protein [46]. Vascular imaging, including CT, MRI, or catheter angiography, may show vasospasm [48]. Brain biopsy remains the diagnostic gold standard [48]. A study examining the incidence of MRI changes among 135 patients with NSCLC receiving ICI therapy found 11 patients with lesions suggestive of ischemic stroke and 4 with lesions suggestive of CNS vasculitis or encephalitis, though other clinical and paraclinical parameters were not reported [49]. 

### 2.2. Peripheral Nervous System Complications

#### 2.2.1. Radiculopathies and Neuropathies

A study including 920 patients treated with ICI estimates the overall incidence of peripheral neuropathy to be 1.2% [50]. Variable phenotypes have been reported, including isolated polyradiculopathy, inflammatory polyradiculoneuropathy (AIDP, CIDP), cranial neuropathies, small-fiber neuropathy, length-dependent polyneuropathy, mononeuritis multiplex, and neuralgic amyotrophy [51,52]. Of these, the most common manifestation is acute polyradiculoneuropathy [12,50]. Clinical presentation is similar to that of idiopathic forms, though a preceding infectious prodrome is uncommon; however, diarrhea secondary to gastrointestinal irAEs has been reported [53]. Cases of Miller–Fisher and anti-Gq1B syndrome have also been reported [12,35]. The median time to nadir from symptom onset was 3.5 weeks in one series [50], though cases of CIDP have been reported [54]. Symptoms may consist of either symmetric or asymmetric sensory and/or motor abnormalities, and autonomic dysfunction may also be observed. Pain in the low back or thighs may be a heralding symptom.

CSF shows elevated protein in most patients, with or without white blood cell elevation [12,50]. Ganglioside and onconeural autoantibodies are usually negative [12,35]. Electrodiagnostic studies typically show changes of an acquired demyelinating polyradiculoneuropathy, with or without secondary axonal loss [51,55]; a minority of patients have subclinical evidence of concurrent myopathy [12,51]. Contrary to idiopathic AIDP, corticosteroids are recommended as part of standard treatment [56]. IVIG and plasma exchange may also be considered.

Cranial neuropathies most commonly involve the facial nerve and are typically associated with abnormal gadolinium enhancement on MRI. Oculomotor, abducens, trigeminal, vestibulocochlear, and glossopharyngeal nerve involvement have also been reported [51,57]. Most patients achieve full clinical recovery with corticosteroid treatment and cessation of the immune checkpoint inhibitor [12,50,51].

#### 2.2.2. Myasthenia Gravis

ICI-related myasthenia gravis (MG) frequently overlaps with myositis (further discussed below). It usually presents in patients with no prior history of MG, but cases of MG exacerbation triggered by ICI therapy have been reported as well [50,58]. The median latency from ICI administration to symptom onset was 6.6 weeks in one study [50] and ranged from 6–106 days in another series [59]. The clinical presentation is more fulminant than idiopathic MG, with more than 50% of patients having bulbar or respiratory muscle weakness [12,50]. The presence of thymoma was not found to be correlated with the development of ICI-related MG [59,60]. However, a recent study in patients with thymoma found that patients with thymoma and MG had fewer CTLA-4 positive cells within the tumor compared to thymoma patients without MG, suggesting a possible association between CTLA-4 downregulation and idiopathic MG [61]. Most patients present with the MG-myositis overlap syndrome, and around 80% of patients have concomitant non-neurologic irAEs, particularly myocarditis [50]. Ptosis can arise from either MG or the involvement of the extraocular muscles by myositis and cannot be reliably used to distinguish one entity from another.

Electrodiagnostic parameters are similar to idiopathic MG and frequently show concomitant myopathy. Acetylcholine receptor (AChR) seropositivity occurs in approximately 60% of patients [12]. Anti-MuSK antibodies are rare [60]. It is thought that patients with pre-existing MG or AChR seropositivity are at higher risk of developing an MG flare when exposed to ICIs [28,59]. A retrospective analysis of 137 patients with non-small cell lung cancer receiving ICIs found that patients with pre-existing non-neurologic autoantibodies were at a higher risk of immune-related adverse events [62], supporting the findings of Suzuki et al. [59] that ICIs could precipitate autoimmunity in patients with an underlying predisposition. However, there is one report of a patient with anti-AChR antibodies who tolerated ICI treatment well without the development of MG [63] and another case of mild MG relapse not requiring any specific therapy [64]. Thus, further study is needed to determine the safety of ICI in asymptomatic patients with anti-AChR antibodies and patients with pre-existing MG. Routine AChR antibody screening, prompting closer neurologic monitoring of seropositive patients during ICI treatment, has been recommended [50], though the relative cost–benefit of this approach is not clear. At this time, we recommend vigilant screening and the evaluation of patients who have symptoms suggestive of neuromuscular junction disorder, as well as a low threshold for closer monitoring or hospital admission in this population. 

The majority improve with treatment, though the mortality rate remains high [12,50,60]. Respiratory failure is more common than that in idiopathic MG, and the presence of concomitant MG and myositis is associated with increased risk compared to MG alone [50], consistent with previous evidence that patients with concurrent MG, myositis, and myocarditis have a higher mortality rate compared to those with MG alone [50,58,60]. 

#### 2.2.3. Myositis

Myositis was the most common neurologic irAE in one systematic review, representing 32% of all cases [12,65]. It usually develops at a median of 5–6 weeks after ICI administration [50,65]. The clinical spectrum is variable, ranging from minimally symptomatic hyperCKemia to severe weakness and rhabdomyolysis [12,65]. The pattern of weakness is typically limb girdle, with frequent neck, bulbar, or respiratory muscle involvement either due to primary myositis or concurrent MG [12,66]. In one series, head drop was a common presenting symptom of ICI-related myositis [50]. Cutaneous manifestations suggestive of dermatomyositis can be seen in around 18% of patients [66]. Orbital myositis causing diplopia and restrictive orbitopathy has been reported, sometimes mimicking MG [42,67]. Most cases had clinical or paraclinical evidence of systemic myopathy [64]. 

Myositis-associated antibodies are usually negative [68,69], though in one review, anti-striational antibodies were present in approximately 50% of patients. MRI typically shows evidence of myositis. Electrodiagnostic studies show changes consistent with an irritable myopathy in approximately 80% of patients [12]. Skeletal muscle biopsy shows necrotizing myopathy in the majority of cases [12,68]. Assessment for concurrent MG with repetitive nerve stimulation is recommended. Electrodiagnostic studies may be used to target specific muscles most amenable to biopsy. In addition, all patients should undergo serum troponin measurement, electrocardiogram, and echocardiography to screen for myocarditis. Cardiac MRI is more sensitive than echocardiography in this situation and should be pursued if the echocardiogram is negative. 

Approximately 70% of patients improve with treatment, though there is a 17% mortality rate [12]. Patients with concurrent MG and myocarditis had a 13.75 higher odds of death compared to those with isolated myositis, consistent with other studies describing a high mortality rate with the so-called “triple M syndrome” [50,60,66]. First line treatment is typically with corticosteroids (e.g., prednisolone 0.5–1 mg/kg/day); a suggested approach is continuing steroids for 4–8 weeks followed by a taper over several months (depending on initial symptom severity) [70]. However, data on optimal duration and tapering schedules are scarce.

## 3. Clinical Approach to Suspected ICI-Related Neurotoxicity

### 3.1. Approach

The presence of concurrent cancer in patients receiving immunotherapy renders them vulnerable to a breadth of pathologies that can affect the nervous system. The differential diagnosis in patients with a suspected neurologic irAE is therefore very broad and includes parenchymal or leptomeningeal metastasis, infections, toxic-metabolic disturbances, paraneoplastic syndromes, and treatment-related effects.

An accurate history of prior cancer treatments, including chemotherapy, radiation, or surgical interventions, is essential. The type of immunotherapy, temporal relationship between drug initiation and symptom onset, and last dose must also be clarified, as the clinical presentation can vary depending on the drug(s) used [11]. The majority of patients present within 4–6 months of starting therapy, usually within the first 4–8 weeks [11,71]. History of prior autoimmune disease, previous paraneoplastic syndrome (PNS), or symptoms of non-neurologic irAEs should also be obtained, as this may indicate an underlying predisposition to autoimmunity [71]. 

**Figure 1:**
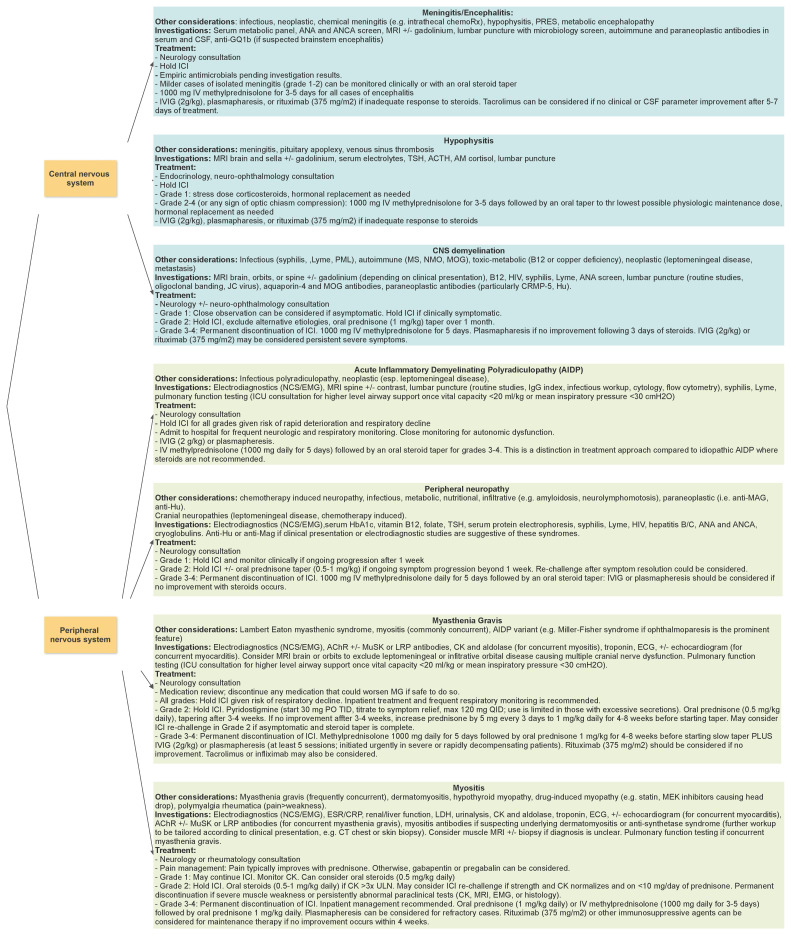
Neurological toxicities of immune checkpoint inhibitors, paraclinical testing, and management [56,72].

A neurological evaluation, preferably by an experienced neurologist, is recommended in order to accurately localize and characterize the clinical symptoms. Figure 1 summaries the diagnostic considerations, investigations, and management for common neurologic irAEs [56,72]. Particular attention should be paid to distinctive characteristics of neurologic irAEs compared to their idiopathic forms, such as the frequent association of MG and myositis in patients receiving ICIs (and the additional risk of concurrent myocarditis in this population). Baseline neurologic examination should also be performed in any patient with a known neurologic disorder prior to initiating immunotherapy. A thorough systemic examination is also warranted to look for signs of other non-neurologic irAEs [71].

Investigations should be tailored to the clinical presentation, though consideration of multiple concurrent syndromes should be considered, as multiple irAEs may occur in a single patient. Patients with CNS symptoms often warrant MRI brain with and without contrast and lumbar puncture, with assessments of cell counts, protein, glucose, microbiology studies, oligoclonal banding and IgG index, and cytology +/− flow cytometry. Both CSF and serum paraneoplastic autoantibody testing using high-quality assays is recommended, especially when the clinical picture resembles a medium or high-risk PNS [71]. EEG is recommended in patients with suspected seizures or encephalopathy. Patients with neuromuscular disorders should be evaluated with nerve conduction studies and electromyography. In the case of polyneuropathy, alternative causes should be sought by checking hemoglobin A1C, vitamin B12, TSH, HIV, syphilis, and protein electrophoresis. An MRI of the spine is recommended to look for mass lesions or abnormal spinal cord or nerve root enhancement. 

The management of suspected neurologic irAEs should begin with holding the ICI until the diagnosis is confirmed. The symptoms are then generally graded by severity, from asymptomatic or mild symptoms (grade 1), moderate symptoms (grade 2), severe (grade 3), to life-threatening (grade 4) [56]. Mild symptoms such as minimally symptomatic aseptic meningitis or peripheral neuropathy can often be managed by temporarily holding the drug and monitoring clinically for symptom resolution. As the majority of demyelinating events due to ICI are monophasic [25], it may be reasonable to continue immunotherapy in asymptomatic patients with neuroimaging evidence of demyelinating lesions and promptly discontinuing the drug if any worsening occurs. 

For moderate symptoms (grade 2), oral prednisone (0.5–1 mg/kg) for 3–4 weeks followed by a slow taper is recommended, except in the case of MG, AIDP, or encephalitis, where IV methylprednisolone should be promptly initiated [56,72]. In addition, IVIG or plasmapheresis should be initiated in all patients with AIDP or MG, regardless of severity, given the risk of rapid decline and respiratory compromise. Severe neurologic irAEs (grade 3–4) should also be treated with IV methylprednisolone followed by an oral steroid taper. IVIG or plasmapheresis should be considered in the acute phase if no improvement occurs with IV steroids. In the case of encephalitis with positive paraneoplastic antibodies, long-term immunosuppression is recommended [56,71,72]. Decisions around the reinstitution of ICI therapy often involve a discussion of relative risks and benefits of rechallenge in the context of the individual patient’s cancer, neurologic syndrome, ongoing neurologic symptoms, and goals of care.

### 3.2. Unanswered Questions

#### 3.2.1. ICIs in the Context of Pre-Existing Autoimmune Disease

In patients with pre-existing systemic autoimmune disease, evidence from prospective studies regarding the risk of irAEs is mixed, with some prospective cohorts demonstrating no significant difference in irAEs in patients with or without prior autoimmune disease and other studies showing a significantly higher number of irAEs among patients with known autoimmunity [73]. No such prospective studies have been performed in patients with a prior history of neurologic autoimmune disease. However, retrospective case series have demonstrated that patients with MG or MS may be at a higher risk of neurologic irAEs following ICI administration [31,58,59], concordant with evidence that patients with rheumatoid arthritis, polymyalgia rheumatica, and psoriasis also have a higher risk of irAEs [73]. Further studies are needed to answer this clinical question.

#### 3.2.2. ICIs and Paraneoplastic Syndromes

Paraneoplastic syndromes (PNS) are thought to occur from spontaneous immune response to ectopically presented neuronal antigens on tumor cells [74]. Experimental mouse models suggest that ICIs may enhance or misdirect the anti-tumor immune response, leading to PNS [74]. 

In a recent retrospective study and review [75], 11/86 patients with neurologic irAEs had positive anti-Hu antibodies following ICI treatment. Compared to ICI-naive patients with anti-Hu seropositivity, the clinical presentations were not significantly different. They also report on five patients with known anti-Hu antibodies prior to ICI administration, all of whom had significant worsening of their PNS following ICI therapy [75]. Another retrospective cohort in France reported an increased frequency of anti-MA encephalitis associated with the increased use of ICIs in a single tertiary center [76]. Multiple other case reports note similar associations between ICI use and de novo or relapsing PNS [77,78,79,80,81]. These findings support the theory that ICIs potentiate misdirected anti-tumor responses. However, there are no prospective studies examining the risk of PNS in patients with pre-existing onconeural antibodies. 

Expert recommendations suggest classifying paraneoplastic syndromes as grade 3 or 4 irAEs and treating them as such [74]. However, it should be noted that PNS directed against intracellular neuronal antigens respond poorly to immunotherapy, and the potential impact of such immunosuppression on underlying cancer progression is unclear [74].

#### 3.2.3. Rechallenging ICI

Prospective studies examining the safety of ICI reintroduction following an irAE are limited. In general, it is estimated that approximately 30–50% of patients may have a recurrent irAE, though the majority were milder than the initial event [82,83,84]. No prospective data exist for neurologic irAEs, but the analysis by Dolladille et al. [84] found that patients with neurologic irAEs did not have a higher recurrence rate following re-challenge compared to other systemic irAEs, with an approximate 6.9% relapse rate. However a large proportion of their rechallenge data did not report the clinical outcome, which likely skews this finding. Therefore, the decision to restart treatment should be made on a case-by-case basis depending on the severity of the initial neurologic toxicity, extent of recovery, and status of their underlying cancer. Patients should also be closely monitored for any signs of recurrent irAEs. Due to the absence of high-quality data to guide decision making and the risk of disability or death, it is recommended that ICI re-challenge is avoided in patients with severe neurologic irAEs (grades 3–4) or any severity of encephalitis, AIDP, or MG [56,72].

## 4. Immune Effector Cell Therapies

### 4.1. Immune Effector Cell Neurotoxicity Syndrome (ICANS)

#### 4.1.1. Epidemiology

Initial CAR T-cell clinical trials were noted to have high rates of neurological symptoms such as encephalopathy [85]. Neurological toxicity, initially called CAR-T cell-related encephalopathy syndrome (CRES) and more recently termed immune effect cell neurotoxicity syndrome (ICANS), is one of the most common adverse events seen with CAR T-cell therapies [85]. It is best characterized in the context of CAR T cells targeting CD19 (an antigen expressed on B lymphocytes and certain B cell lymphomas/leukemias) [85]. The published incidence of ICANS varies from 37–77% as per a recent systematic review of the literature primarily evaluating CD19 products [85,86], though a recent phase 3 trial of tisagenlecleucel reports a much lower incidence (10% incidence of any neurological event) [87]. The incidence of high-grade neurotoxicity in patients treated with CAR T cells in clinicals trials is highly variable, partially due to variable definitions being employed prior to the standardized grading of ICANS, with reported ranges between 0 and 45% of treated patients [88,89]. 

#### 4.1.2. Risk Factors for ICANS

Features associated with an increased likelihood of developing ICANS include a high baseline tumor burden [90], greater CAR T cell expansion [85], an earlier and more severe systemic inflammatory response called cytokine release syndrome (CRS) (with high peak ferritin and C-reactive protein levels) [91,92,93], and chimeric receptors with a CD28 co-stimulatory signaling domain [85,94,95]. CD19-targeted CAR T cells have thus far been associated with more neurotoxicity than CD20, CD22, or B-cell maturation antigen (BCMA)-targeted CAR T cells [95,96]. A recently published phase 3 study of Ide-cel, a BCMA-targeted CAR T cell product, in multiple myeloma reported neurotoxicity in 15% of patients. CRS was seen in 88% of patients, nearly all low grade [97], compared to a historical incidence of 43–77% in CD19 products used to treat B-cell lymphomas [86]. Furthermore, patients infused with higher cell counts of CAR T cells are more likely to develop ICANS (and CRS).

#### 4.1.3. Suspected Pathophysiology

Interleukin-1 (IL-1) is thought to be an essential cytokine upstream of macrophage activation and interleukin-6 (IL-6) release. The latter is believed to be the major effector of CRS [98,99]. While there is certainly an association between CRS and ICANS and the latter is rarely seen without being preceded by the former, the exact mechanism of ICANS is not understood. Increased permeability of the blood–brain barrier likely plays a role. Astrocytic injury may contribute to this, as increased CSF levels of markers of astrocytic injury are seen in patients with ICANS [100]. Endothelial activation with increased vascular permeability may contribute [95,101]. IL-6 may play a role given that higher peak IL-6 serum levels are seen in ICANS patients compared to those who do not develop neurological symptoms [91]. IL-6 and certain other cytokines (such as IFN-gamma and IL-10) are also seen in the CSF of patients with neurotoxicity [100]. Interestingly, treatment with tocilizumab, an IL-6 receptor inhibitor, is effective for CRS but not ICANS. However, given the poor penetrance of tocilizumab across the blood–brain barrier, paradoxically, it could increase the concentration of free IL-6 to which the central nervous system is exposed [95].

The infiltration of CAR T cells into brain parenchyma was not seen in autopsy from a fatal case of ICANS with cerebral edema [102], suggesting that, at least in cerebral edema, direct infiltration by CAR T cells was unlikely to be the mechanism for neurotoxicity leading to cerebral edema.

#### 4.1.4. Clinical Presentation

The term ICANS applies broadly to central nervous system pathology attributed to treatment with immune effector therapies [103]. ICANS consists of a spectrum of neurological symptoms ranging from headache to life-threatening or fatal cerebral edema thought to be caused by an underlying systemic cytokine response. Neurological symptoms most commonly seen in ICANS include headache, tremor, and confusion, with respective incidences of 25%, 13%, and 10% as per a 2021 meta-analysis [104]. Most common neurological symptoms of grade 3 or higher severity include seizure (11%), aphasia (17%), and encephalopathy (28%) [104]. While many of the symptoms seen in ICANS can be seen in a variety of disorders, aphasia is thought to be the most specific early finding in ICANS, ranging from dysgraphia to global aphasia. Thirty-five percent (35%) of patients had language disturbance in one cohort [91]. In most, decreased fluency or word finding difficulties were early presentations. This typically progressed over hours to days, and patients typically also developed encephalopathy. A subset of patients developed severe aphasia, some becoming mute and globally aphasic. Encephalopathy can have a broad range of severity, from mild inattention to agitation or lethargy and a decreased level of consciousness [91]. Although less common, apraxias have been described as well, including ideomotor apraxia in the absence of encephalopathy [91], as well as general executive disorders, dyscalculia, and non-lesional neglect [92]. Movement disorders are also commonly noted. Mild increased physiological tremor is most frequent (28% in one cohort) and is often an early sign [91]. Other tremor types have also been seen, as well as myoclonus, asterixis, and cerebellar findings. Focal weakness is rarely seen, in some cases associated with an underlying structural cause, particularly stroke or hemorrhage, but sometimes not having a structural cause identified and resolving as ICANS improved [91]. The most severe cases of ICANS result in seizure, even status epilepticus, or death from cerebral edema [100,102].

#### 4.1.5. ICANS Timing and Relationship to Cytokine Release Syndrome

ICANS is often preceded by CRS in at least 80–90% of ICANS cases, and this association is likely even higher in severe cases of ICANS. Some cohorts report all ICANS being preceded by CRS [91,92,101]. CRS is a systemic syndrome defined by fever with or without hypoxia and hypotension ranging in severity from mild symptoms to requiring intensive care support for respiratory support or vasopressors [103]. While there is clearly a relationship between CRS and ICANS, they are considered distinct entities [103].

ICANS typically presents about seven days after CAR T-cell infusion [87,89,92] but can likely occur from hours to three weeks after CAR T-cell infusion. It typically resolves over weeks, with a mean duration of about 10 days [86,89,105]. In one cohort, the median time from fever (and CRS) onset to the first neurological symptoms was about 4.5 days, ranging from 2 days to nearly 3 weeks [101].

#### 4.1.6. Paraclinical Testing

Neuroimaging appears to be normal in most ICANS patients, with many reported case series of ICANS reporting unremarkable MRIs in all patients [86,92]. However, some patients, particularly those with high-grade ICANS, can have diverse findings on MRI. Reported findings include T2 FLAIR hyperintensities, particularly of deep gray matter, and leptomeningeal enhancement or subcortical edema suggestive of posterior reversible encephalopathy syndrome. Ischemic stroke and multifocal microhemorrhages have been reported in severe cases, as well as cortical laminar necrosis. Signs of cerebral edema, the hallmark of the most severe cases of ICANS, are rarely seen [86,106]. These imaging abnormalities are mostly reported as case reports or small case series and so their true incidence is difficult to estimate but is likely quite low. In this context and given the significant systemic comorbidities and malignancies that could confound some of these findings, alternate etiologies should be carefully ruled out in patients with abnormal MRI findings.

The most common electroencephalogram (EEG) finding in ICANS is generalized slowing, which appears to correlate with the severity of ICANS (seen in 28 of 36 ICANS patients in one study), while focal slowing is also common (12 of 36 patients) [91]. Epileptiform discharges were rare (3 of 36 patients), while one patient had a clinical generalized tonic clonic seizure in this cohort. In a different cohort of 81 patients with ICANS, about half of the patients had rhythmic patterns on EEG, while a minority of patients were found to have clinical or electrographical seizures [107]. 

Laboratory findings associated with ICANS typically pertain to the CRS and underlying malignancy, with cytopenias being quite common as well as high inflammatory markers (particularly C-reactive protein and ferritin). While certain cytokines can also be elevated, these are not typically followed clinically [100,108]. Data from CSF are limited given the frequency of cytopenias, particularly in the most affected patients. Furthermore, CSF studies are not typically done on patients treated with CAR T cells in the absence of neurological symptoms, and so it is difficult to interpret results from ICANS patients. Overall, CSF can be abnormal, often showing increased protein and pleocytosis, which can vary significantly in severity, as well as high levels of different cytokines that are not tested routinely in clinical practice [91,92,101].

#### 4.1.7. Grading

**Figure 2:**
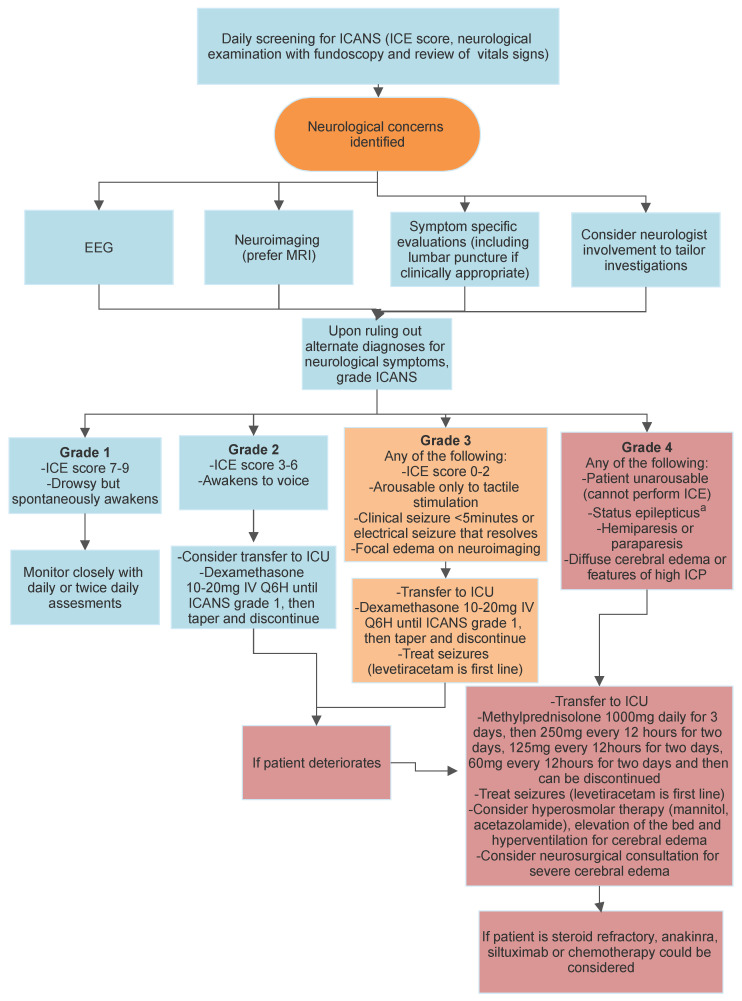
Paraclinical testing, grading, and management of ICANS.

Early CAR T-cell literature was confounded by the use of inconsistent nomenclature and grading systems for both CRS and ICANS. Since 2018, a standardized grading system was proposed by the American Society for Transplantation and Cellular Therapy (ASTCT), which has led to better consistency amongst studies [103]. The grading of ICANS is based on a patient’s score of their performance on a short battery of tests focused on language, orientation, and attention (Immune effector Cell Encephalopathy (ICE) score, described in Table 1), as well as their level of consciousness, whether they have had any seizures or signs of raised intracranial pressure on imaging, and whether they have any focal weakness (see Figure 2). The highest grade ICANS (grade 4) is defined by the patient being unarousable, being in status epilepticus, having new hemiparesis, or clinical or imaging findings worrying for life-threatening elevation in intracranial pressure. Fundoscopy is often recommended to identify the presence or absence of papilledema, which can be informative in some grading schema.

#### 4.1.8. Clinical Screening for the Presence of ICANS

Routine screening for the presence of ICANS should be performed for all patients receiving CAR T-cell therapy or blinatumomab. A baseline neurologic examination should be performed on all patients planned for therapy so that their neurologic baseline is established. A daily clinical evaluation including the following should be performed: general systemic evaluation and review of vital signs, ICE score, and a general neurologic examination, which includes fundoscopy [109,110].

The ICE score was derived from the assessment of five clinical domains: orientation, naming, following commands, writing, and attention [103]. The scoring tool was derived from consensus recommendations in an effort to provide a tool that was easy to apply and could better characterize ICANS in the clinical setting and in future clinical trials. 

#### 4.1.9. Management

ICANS should be suspected in patients with neurological symptoms occurring hours to three weeks after CAR T-cell infusion, particularly in individuals in whom symptoms are preceded by cytokine release syndrome. A proposed algorithm for the investigation and management of such patients is described in Figure 2. 

After treatment with CAR T-cell products, patients should be monitored carefully for the appearance of CRS and ICANS symptoms [103]. Intensive care unit admission is recommended for grade 3 or 4 neurological toxicity [110]. Given that patients receiving CAR T cells typically have active malignancies and cytopenias, they are at particularly high risk of complications related to their malignancies as well as infection and hemorrhage [96]. As such, upon the appearance of neurological symptoms, patients should be appropriately investigated (depending on the presenting symptoms) to exclude alternative diagnoses as well as to grade ICANS [103]. EEG is recommended in all patients with suspected ICANS to evaluate for nonconvulsive seizures [107], as seizure can contribute to the altered awareness, aphasia, or impaired attention seen in ICANS.

Appropriate imaging should be done for patients with ICANS [111]. A non-contrast CT scan may be used in a patient with rapid clinical deterioration to evaluate cerebral edema or a structural lesion. MRI is the preferred imaging modality for most patients with clinical deterioration from ICANS. As previously described, MRI will be normal in most patients with ICANS but is needed to exclude alternate etiologies or structural correlates of severe ICANS, such as cerebral edema. Lumbar puncture should be considered, particularly to exclude infectious etiologies, but may not be possible given cytopenias and possible cerebral edema [96]. Neurological consultation is recommended to assist in evaluating for potential alternate etiologies and in managing symptoms of ICANS. 

Intravenous steroids are often the first line of therapy, and tocilizumab is used only in the presence of concomitant CRS as it does not significantly penetrate the blood–brain barrier. As with any patient with neurological symptoms, appropriate measures should be taken for the prevention of aspiration, including elevating the head of the bed and routinely evaluating whether patients can safely swallow pills and food [96]. Seizures should be managed with antiepileptics and benzodiazepines as needed if these occur, with levetiracetam being the most commonly used antiepileptic in ICANS. Some guidelines recommend starting levetiracetam (750 mg twice daily) to prevent seizures at the onset of neurological symptoms, though the utility of this is debated [86,105,110]. Signs of increased intracranial pressure should prompt the urgent involvement of a neurologist and the consideration of neurosurgical consultation [112]. Treatments such as acetazolamide (1000 mg IV once and then 250 mg–1000 mg IV every 12 h thereafter) and hyperosmolar mannitol are recommended. Routine measures for the management of increased intracranial pressure should be undertaken urgently, including elevating the head of the bed and hyperventilating the patient [110]. Patients with severe ICANS should be monitored in an intensive care unit setting. 

CRS is generally managed depending on severity, with IL-6 antagonists such as tocilizumab and siltuximab and/or corticosteroids [112]. As for ICANS, exact recommendations are somewhat varied in the literature. ICANS grade 2 or higher warrants treatment with intravenous dexamethasone or intravenous methylprednisolone, followed by a steroid taper [109,110]. As per JH Rees [110], high-dose intravenous dexamethasone (10 to 20 mg every 6 h) is recommended for grade 2 and 3 ICANS until ICANS improves to grade 1, and then dexamethasone can be tapered and discontinued. Grade 4 ICANS should be treated with intravenous methylprednisolone (1000 mg daily for 3 days, then 250 mg every 12 h for two days, 125 mg every 12 h for two days, 60 mg every 12 h for two days, and then can be discontinued) (Figure 2) [110,113]. The ideal duration of steroids is unknown, though, reassuringly, in a small cohort, 7–10 days of treatment was not associated with worse outcomes compared to fewer than 7 days of steroid treatment [114].

No current consensus exists regarding the treatment of severe ICANS that is unresponsive to steroids. Different strategies to modulate CAR T-cell activity or even kill CAR T cells have been employed. Amongst other potential treatments, there may be a role for anakinra (an IL-1 receptor antagonist that targets the signaling pathway that is likely upstream of IL-6 production) or siltuximab (a monoclonal antibody that targets IL-6 directly) [110,112].

#### 4.1.10. Prognosis

Deaths due to ICANS are uncommon and are typically due to cerebral edema or complications of disseminated intravascular coagulation. There are minimal long-term data regarding the neurological health or cognition of patients having had ICANS. However, prognosis in most is favorable, particularly if recognized and treated appropriately [10,115]. Most patients will return to their neurological baseline by 2 months [92]. 

The question of whether this population has longer-term treatment toxicities, including neurocognitive toxicities, remains an ongoing question. Few studies have followed patient reported outcomes after CAR T-cell therapy. Ruark et al. [116] studied 40 patients who were at least 12 months post CAR T-cell therapy. Self-reported residual cognitive complaints in this population were seen at a similar rate compared to what is reported after standard chemotherapy or stem cell transplantation. The rates of reported depression, anxiety, and fatigue in this population were also similar to those of the general population. Barata et al. [117] studied self-reported cognitive changes in 118 patients having been treated with CAR T cells over 360 days. They noted a subtle worsening in perceived global cognition, with worse self-reported cognition on day 360 in patients having had severe ICANS. Depression and anxiety have been reported in patients who have recovered from neurotoxicity, particularly older patients with less psychosocial support [118]. Further studies of the long-term cognitive and neuropsychiatric status of patients treated with CAR T cells are clearly warranted.

#### 4.1.11. A New Avenue: Emerging Off-Tumor On-Target Effects

BCMA-targeted CAR T cells have been introduced into clinical practice in recent years in the context of multiple myeloma. While the incidence of ICANS appears lower with these products, new rare toxicities have appeared. Emerging data suggest that a rare movement disorder and neurocognitive syndrome can be associated with BCMA-targeted CAR T cells [119]. An early description of this syndrome suggests that it is phenotypically similar to Parkinson’s disease (with bradykinesia, asymmetrical action and rest tremor, postural instability, hypophonia, and saccadic intrusions with impaired memory). However, the characterized cases are levodopa unresponsive. A single case study showed caudate hypometabolism on FDG-PET/CT with infiltration of the caudate by CAR T cells found on autopsy. This was thought to be due to low-level expression of the BCMA antigen expressed in the caudate [120]. Although the mechanism appears to be different from that of ICANS, patients with this syndrome were also more likely to have had cytokine release syndrome, a high tumor burden, and high proliferation of their infused CAR T cells [119]. 

Most recently, a dose-escalation study for a CAR T-cell product targeting another antigen seen in multiple myeloma cells, G-protein-coupled receptor, class C, group 5, member D (GPRC5D), suggests that a small number of patients may develop cerebellar complications with this product [121]. These examples illustrate the potential for off-tumor neurological complications that can be varied in nature. To date, experience with off-tumor neurological toxicities is quite limited, and no consensus has been reached regarding treatment. In severe cases, chemotherapy such as cyclophosphamide has been used to ablate causative CAR T cells [120]. Surveillance to detect such complications and treat them appropriately will be of paramount importance moving forward.

### 4.2. Bispecific T-Cell Engagers (BiTEs)

While ICANS has been best characterized in the context of CD19-targeted CAR T-cell therapies, a similar neurological toxicity has emerged with treatment with bispecific antibodies, particularly blinatumomab, a bispecific CD3/CD19 T cell engager. Both a cytokine-release syndrome and similar neurological symptoms as seen in CAR T-cell-related ICANS (headache, encephalopathy, altered mental status, aphasia, tremor, and seizure) have been described in patients receiving blinatumomab [122]. While the appearance of neurological symptoms is most likely to appear at the time of the first blinatumomab administration (most likely within the first two weeks of initiating blinatumomab) [123], it can also occur during any time at which blinatumomab is administered. About 50% of the patients in a phase II study of 189 patients being treated with blinatumomab had neurological symptoms [124]. A meta-analysis of safety data amongst a total of 885 patients receiving blinatumomab calculated an incidence of 7% of neurotoxicity of grade 3 or higher [122]. Indeed, neurotoxicity and severe CRS are also the most likely adverse events to result in treatment discontinuation or death. The risk of neurotoxicity is higher when blinatumomab is used with concomitant intrathecal chemotherapy, which is not an infrequent situation in acute leukemia treatment, particularly in the setting of CNS involvement of disease [125]. Neurotoxicity also appears to be dose dependent [125].

Neurotoxicity associated with blinatumomab should be treated in a similar fashion to ICANS seen with CAR T cells, with steroids being the cornerstone of treatment [126]. Elements specific to blinatumomab include recommendations to pretreat with dexamethasone in an effort to prevent CRS and to increase dosing in a stepwise and gradual fashion. For grade 3 and higher neurotoxicity, blinatumomab should be discontinued until neurotoxicity is resolved or grade 1 for at least 3 days, and patients should receive dexamethasone. It can then be resumed at a lower dose. Permanent discontinuation is recommended for patients with grade 4 neurotoxicity or grade 3 neurotoxicity lasting at least 7 days or recurring with the reintroduction of the drug [126,127].

## 5. Conclusions

As the indications for cancer immunotherapy expand, the recognition of potential irAEs is essential in order to accurately diagnose and manage patients. The clinical presentation is heterogenous, but there are several distinct clinical patterns of neurologic irAEs that are becoming more evident with time. However, evaluation by a neurologist is recommended in order to characterize the syndrome and exclude other potential etiologies. 

There remains much to be elucidated in terms of understanding the pathogenesis and prognostic markers. Other research questions include the impact of immunosuppressive treatment on the underlying tumor and the efficacy of immunotherapy, the risk of pre-existing paraneoplastic autoantibodies for the subsequent development of neurologic irAEs or paraneoplastic syndromes, and optimal management regimens and the safety of rechallenge in patients with neurologic toxicity.

Increasing recognition of ICANS and treatments to decrease the severity of CRS will hopefully improve the safety of immune effector therapies. However, treatment paradigms for the rare cases of steroid-unresponsive ICANS will need to be developed. Newly emerging biomarkers of neuronal toxicity such as neurofilament [128,129] may become useful in the prompt identification and treatment of patients with ICANS, but their utility remains to be assessed. The development of models to predict who is at high (or low) risk would help the community better tailor patient therapy and potentially predict who could safely be discharged home after treatment with cell therapies [93]. While we continue to develop expertise in the context of the clinical use of CAR T cells and bispecific antibodies targeting CD19-positive cells, we have less clinical expertise with new targets such as BCMA. Novel off-tumor on-target neurotoxicities have emerged recently in the context of non-CD19 targets. Surveillance and further characterization of these will be important moving forward.

While this review has focused on adverse events related to immune therapies in oncology, in many cancer types, these new immune therapies offer remarkable survival benefits. As such, we do expect that the indication for immune therapies will continue to grow, emphasizing the importance for neurologists, oncologists, and the medical community as a whole to be aware of these important potential complications.

## Figures and Tables

**Table 1:** Immune Effector Cell-Associated Encephalopathy (ICE) Score [103].

Domain	Allocated Points
Orientation to year, month, city, hospital	4
Naming of three objects	3
Following a command	1
Writing a standard sentence	1
Attention (counting backwards from 100 by 10)	1
Total number of points	10

## Data Availability

Data sharing not applicable No new data were created or analyzed in this study. Data sharing is not applicable to this article.
